# What We Owe to Experiments on Animals

**Published:** 1903-12-19

**Authors:** Stephen Paget


### Hospital Clinics and Medical Progress.

### WHAT WE OWE TO EXPERIMENTS ON ANIMALS.

By Stephen Paget.

### *I.—PHYSIOLOGY.*

(*Continued from page* 186.)

### VII.—The Nervous System.

The experiments made by Galen on the nervous system were centuries ahead of their age; and after him men neglected the method that he had followed, and were content to worship his name. The whole physiology of the nervous system was founded again, early in the nineteenth century, by the work of Sir Charles Bell and Magendie. In 1811 Bell issued his famous pamphlet, "An Idea of a New Anatomy of the Brain, submitted for the observation of the author's friends." In it he describes how he discovered the difference between the anterior (motor) nerve-roots and the posterior (sensory) nerve-roots. The discovery of this difference between the two sets of nerve-roots was the starting-point of the whole physiology of the nervous system. We are not concerned with the question of priority between Bell and Magendie, for they both of them got their results by the help of experiments on animals: but we are concerned, and very much concerned, with the fact that Bell, in his 1811 pamphlet, says emphatically that experiments on animals were the means whereby he made this great discovery.

For, twelve years later, and again seven years later still, he wrote in a very different way about experiments in general, and his own in particular. If anybody cares to study this curious inconsistency, and the causes of it, he must be referred to the book entitled "Experiments on Animals" (John Murray. 1903). But, after all, Sir Charles Bell has been dead more than sixty years; he lived before anæsthetics, before antiseptics, before bacteriology; and, if he were alive now, he would rejoice to see what results have been won since his time. Now for his own account of his discovery, written at once after he had achieved the whole thing:—

"I found that injury done to the anterior portion of the spinal marrow convulsed the animal more certainly than injury to the posterior portion; but I found it difficult to make the experiment without injuring both portions.

"Next, considering that the spinal nerves have a double root, and being of opinion that the properties of the nerves are derived from their connections with the parts of the brain, I thought that I had an opportunity of *putting my opinion to the test of experiment*, and of *proving* at the same time that nerves of different endowments were in the same cord (nerve-trunk) and held together by the same sheath."

"On laying bare the roots of the spinal nerves, I found that I could cut across the posterior fasciculus of nerves, which took its origin from the posterior portion of the spinal marrow, without convulsing the muscles of the back; but that on touching the anterior fasciculus with the point of the knife, the muscles of the back were immediately convulsed."

"*Such were my reasons* for concluding that the cerebrum and cerebellum were parts distinct in function, and that every nerve possessing a double function obtained that by having a double root. *I now saw the meaning* of the double connection of the nerves with the spinal marrow; and also the cause of that seeming intricacy in the connections of nerves throughout their course, which were not double at their origins."

In the same way he made his discovery that the fifth cranial nerve contains both motor fibres and sensory fibres, and that the facial nerve has nothing to do with facial neuralgia.[1] It is absolutely certain, from his own account of his own work, that his discoveries were the direct and immediate result of experiments on animals. Later, his dislike of Magendie, and his love of anatomy above physiology, led him into a vain attempt to explain away this obvious fact.

Marshall Hall (1790–1857), by his experiments on the spinal cord, discovered the principles of reflex action. Thirty years before him, Prochaska, by the help of similar experiments, had shown that the cord does not only transmit impulses but also generates them; but Marshall Hall proved that these generating stations in the cord are definite structures, each having its appointed place and its own particular work; that is to say, he discovered the spinal centres, the departmental or segmental nature of the cord, the localisation of the sources of reflex action. In 1832–33 he thus demonstrated the reflex movements of the pharyngeal, laryngeal, and sphincter muscles: in 1837, the passage of impulses from one generating-station to another, the effects of drugs on these centres in the cord, and the reflex nature of certain convulsive movements.

Flourens (1794–1867), following him, discovered in the medulla oblongata the centres that govern the movements of the heart and of the respiratory muscles. He also discovered the influence of the cerebellum on the co-ordination of the movements of the body; and thus led the way to the discovery of the action of the semi-circular canals in the maintenance of the sense of equilibrium.

Claude Bernard (1813–1878) published in 1851 his account of the vaso-motor nerves; the first proof that the calibre of the arteries is under the direct control of the central nervous system. His account of this great discovery, perhaps the most important in all physiology since Harvey's time, is best told in his own words:—

"Starting from the clinical observation, made long ago, that in paralysed limbs you find at one time an increase of cold and at another an increase of heat, I thought this contradiction might be explained by supposing that, side by side with the general action of the nervous system, the sympathetic nerve might have the function of presiding over the production of heat. . . . This was a theory, that is

[1] "Up to the time that Sir Charles Bell made his experiments on the nerves of the face, it was the common custom of surgeons to divide the facial nerve for the relief of neuralgia, *tic douleureux*; whereas it exercises, and was proved by Sir Charles Bell to exercise, no influence over sensation, and its division consequently for the relief of pain was a useless operation."—*Sir J. Erichsen.*

to say, an idea leading me to make experiments; and for these experiments I must find a sympathetic nerve trunk of sufficient size, going to some organ that was easy to observe, and must divide this trunk to see what would happen to the heat-supply of the organ. You know that the rabbit's ear, and the cervical sympathetic nerve of this animal, offered us the required conditions. So I divided the nerve, *and immediately my experiment gave the lie direct to my theory.* I had thought that the section of the nerve would suppress the function of nutrition, of calorification, over which the sympathetic system had been supposed to preside, and would cause the hollow of the ear to become chilled; and here was just the opposite, a very warm ear, with great dilatation of its vessels. I need not remind you how I made haste to abandon my first theory, and gave myself to the study of this new state of things. *And you know that here was the starting-point of all my researches into the vaso motor and thermic system;* and the study of this subject is become one of the richest fields of experimental physiology."

Fritsch and Hitzig, in 1870, began the work of mapping out the motor centres on the surface of the brain. "By the use of very weak currents over very small areas, which we will call centres for the sake of brevity," they succeeded, where other men had failed, in starting the work of cerebral localisation. Before 1870, the speech-centres had been recognised, by clinical experience and by post-mortem study, without the help of experiment on animals. But there the advance of discovery had stopped. From 1870 it went forward till it attained a whole world of facts that the older physiologists had never dreamed of.

---

### Summary of Experiments in Physiology.

The foregoing chapters illustrate what Mr. Darwin said in his evidence before the Royal Commission in 1875: "I am fully convinced that physiology can progress only by the aid of experiments on living animals. I cannot think of any one step which has been made in physiology without that aid." The facts are more fully stated in the book "Experiments on Animals" (John Murray, 1903). But many other facts have been passed over, both there and here. "Many examples," says the book, "have been left out altogether—the work of Boyle, Hunter, Lavoisier, Haldane, Despretz, and Regnault, on animal heat and on respiration; of Petit, Dupuy, Breschet, and Reid, on the sympathetic system; of Galvani, Volta, Haller, du Bois Reymond, and Pflüger, on muscular contractility: nothing has been said of the work lately done on the suprarenal glands and 'adrenalin,' and on the blood-pressure in its relation to secretion. For the most part, only those examples have been taken that occur far back in the history of physiology; more has been said about the past than about the present. . . . But the experimental method, alike in the past and in the present, has been the chief way of advance. And if a forecast may be made without offence, it is certain that the work of physiology, as in the past and the present, so in the near future, will exercise a profound influence for good on medical and surgical treatment. Among the subjects that especially occupy physiologists now, are the more exact localisation and interpretation of the special sense centres, and the better knowledge of the internal secretions and chemical influences of the glands and tissues of the body. It would be hard to find two fields of work more sure to favour the growth of the *arbor vitæ* side by side with the *arbor scientiæ.*"

(*To be continued.*)

